# Technological Answerability and the Severance Problem: Staying Connected by Demanding Answers

**DOI:** 10.1007/s11948-021-00334-5

**Published:** 2021-08-24

**Authors:** Daniel W. Tigard

**Affiliations:** Institute for History and Ethics of Medicine, Technical University of Munich, Ismaninger Str. 22, 81675 Munich, Germany

**Keywords:** Technological answerability, Machine ethics, Moral responsibility, Moral agency, Human–computer interaction, Artificial intelligence, Explainable AI, Robot ethics

## Abstract

Artificial intelligence (AI) and robotic technologies have become nearly ubiquitous. In some ways, the developments have likely helped us, but in other ways sophisticated technologies set back our interests. Among the latter sort is what has been dubbed the ‘severance problem’—the idea that technologies sever our connection to the world, a connection which is necessary for us to flourish and live meaningful lives. I grant that the severance problem is a threat we should mitigate and I ask: how can we stave it off? In particular, the fact that some technologies exhibit behavior that is unclear to us seems to constitute a kind of severance. Building upon contemporary work on moral responsibility, I argue for a mechanism I refer to as ‘technological answerability’, namely the capacity to recognize human demands for answers and to respond accordingly. By designing select devices—such as robotic assistants and personal AI programs—for increased answerability, we see at least one way of satisfying our demands for answers and thereby retaining our connection to a world increasingly occupied by technology.

## Introduction

Artificial intelligence (AI) and robotic technologies have become nearly ubiquitous in the lives of many humans today. In some ways, the developments have likely helped us, from providing mundane pleasures like fitting entertainment recommendations to assuring our health and safety, for example, via fitness tracking apps or sophisticated traffic-control signals. In other ways, however, technologies undoubtedly hurt us. There are the more overt harms, like breaches of data privacy and algorithmic threats to autonomy (cf. Véliz, 2020; Wachter & Mittelstadt, 2019). There are also less obvious sorts, such as the long-term effects of human–computer interaction upon society or upon individuals’ psychological wellbeing. Among this latter sort is what has recently been dubbed the ‘severance problem’ (Danaher, 2019a), and indeed it can be traced back to foundational thinkers in the philosophy of technology (e.g. Borgmann, 1984). The central idea is that technologies *sever* our connection to the world, a connection which is necessary for us to flourish and live meaningful lives. In this essay, I grant that the severance problem is a threat we should work to mitigate if we are to live comfortably with today’s emerging technologies. The question, then, is: how exactly can we stave off the threat? What are some ways we might maintain our connection to the world in the face of technologies that might otherwise tear us away from it?

There are, I believe, several promising approaches that have recently been put forward, which can be construed as more or less directly addressing this line of inquiry. Some are as simple as encouraging people to “unplug”—at least periodically—from our technological devices (Rowan, 2010). This might allow us to retain or rediscover an appreciation for analog tools and activities (Sax, 2016). By contrast, some approaches encourage us to plug in *more*, for example, to explore virtual worlds wherein we can develop skills and forge relationships with fellow virtual players (Danaher, 2019a). Depending upon the technology in question, and upon the users, these tactics might succeed in allowing us to harness some of the benefits of technology without being too hurt by it. With this paper, I want to explore and provisionally defend an option that occupies a middle-ground between unplugging and diving deeper into digital worlds. The idea can be referred to as ‘technological answerability’ and it applies to systems like robotic assistants and personal AI programs, devices intended for direct and regular interaction with individual users.

As a response to the severance problem, my overarching argument can be summarized as follows. First, it seems that some technologies sever our connection to the world by failing to provide answers for why certain behaviors are undertaken, or why some outputs are given. But our demands for answers are, in most cases, psychologically important and worth satisfying.^1^ Although we could simply discontinue our use of such devices, instead, we could assure that our demands for answers are satisfiable, at least by select devices. One way to assure that our demands for answers are satisfiable would be to design devices with a degree of technological answerability—namely, the capacity to recognize human demands for answers and to respond accordingly, as I explain in further detail below. With improved opportunities to provide answers, then, sophisticated technologies are less likely to sever our connection to the world.

Undoubtedly, there is much to be unpacked from this summary, and I will begin by expanding upon the so-called severance problem. Here I also establish the sorts of demands I have in mind and how technologies might not be capable of satisfying them. Next, I explain further how our demands are valuable and, thus, why we should work to satisfy them in our interactions with some technologies. I then develop and apply the notion of technological answerability, showing how it might help to accommodate our demands and thereby retain our connection to the world around us. Before closing, I present several qualifications and a looming objection, namely the thought that such technologies risk *further severing* our connection by deceiving us and removing us from decision-making processes. I close with some final thoughts on responsibility and the prospect of staying connected to the world.

## Technological Severance and Demanding Answers

To understand the demands that will be considered, it should help to remark, first, that I have in mind a subset of a wider class of natural human responses. That wider class, in contemporary ethics literature, is often referred to as the ‘reactive attitudes’.^2^ Briefly, we respond naturally to the world, to others with whom we share it and to ourselves, in ways that indicate, or even help to formulate, our moral approval or disapproval. For example, when I am harmed—say, as a result of a friend’s carelessness—I am understandably upset, perhaps disappointed, and so on. These responses are illustrative of the expectations we hold for others and for ourselves (cf. Strawson, 1962; Wallace, 1994). They also depend largely upon our social roles, the rights and duties we ascribe, and our relationships, as I explain further below. Importantly, I can express my reactions in order to elicit another’s recognition and response, like an apology and adjustment of their future behavior. Similar mechanisms are often at work in our experience of positive events: we are pleased by them, we appreciate those who caused them, and so on. The basic point to be made here is that there is a great variety of ways in which we respond to others, notably, ways that serve to locate a sense of moral responsibility.

According to some recent accounts, demanding answers or reasons for others’ actions and decisions is a key mechanism—perhaps even a distinct *type*—of moral responsibility. It is, for David Shoemaker (2015), unlike the notion of accountability, whereby we evaluate the quality of an agent’s regard for others. It is also unlike the ways we attribute to an agent the actions or attitudes that appear to express their underlying character (Shoemaker, 2015; Watson, 2004). Instead, answerability is a process by which we demand reasons and justifications, an answer to the question of *why* an agent behaved in some way or other. In this way, the demand for answers does not evaluate one’s character, or one’s moral regard or lack thereof; but rather, it evaluates an agent’s judgment. In Shoemaker’s words, an agent is answerable “just in case the agent could in principle cite his or her ‘instead of’ reasons” for performing some action (2015, p. 82). I will return to this notion, but first I want to situate my discussion against some of the recent work in technology ethics and show how this sort of responsibility is threatened by our interactions with some devices.

Taking their cue from Andreas Matthias’s momentous essay, some authors have worried that the use of autonomous, learning machines will “create a new situation, where the manufacturer/operator of the machine… cannot be held morally responsible or liable” (Matthias, 2004, p. 175). Developers and users of some of today’s technologies do not have sufficient knowledge or control to be appropriately considered responsible for the actions and outcomes brought about. As such, the so-called ‘responsibility gap’ stands to undermine our moral and legal notions of responsibility.^3^ My attention will remain on moral notions, and as suggested, I will focus on a subset of those responses. That is, I am less concerned here with the question of accountability in technological systems.^4^ Indeed, in a strictly moral sense, it will be difficult to hold anyone (or anything) to account for technological harms, considering that very often no one really *deserves* our accountability responses, like retribution via punishment (cf. Danaher, 2016a; Sparrow, 2007). Equally, I am less concerned here with the process of attributing harm to technological systems, since it seems at least intuitively implausible to think a machine’s behavior could express its underlying character.

My main concern is the prospect of holding technological systems, such as AI and robotic devices, *answerable* for their conduct. And, admittedly, I do not think we will neatly find this sort of responsibility in technology itself, since, like other sorts of moral responsibility, being truly answerable is tied to distinctly human capacities and interactions. In other words, responsibility is often predicated on the robust *natural* sort of agency we find only in creatures like ourselves.^5^ Since technological entities cannot possess the fullest sense of agency we enjoy, it is commonly thought that they cannot really be responsible. This is why most authors seek out modes of responsibility in designers, developers, companies, users, regulators, and so on. For otherwise, we may be forced to accept that there is a “gap” in responsibility. However, I do not want to settle for a process of seeking answers only from a system’s human associates, such as designers, users, or collections, as others suggest (e.g. Coeckelbergh, 2020; Nissenbaum, 1996; Nyholm, 2018, 2020; Rahwan, 2018). Instead, I set my sights on technology itself, but not because I agree with those who find there are no human associates who deserve responsibility. In fact, it seems there are often good reasons to *not* let humans off the hook—or for someone to “take” responsibility where they would otherwise be free of blame (cf. Mason, 2019; Tigard, 2019). Rather, I want to explore the prospect of holding technology itself responsible because the systems, devices, and apps themselves are what increasingly occupy our everyday lives, not the human developers and lawmakers who create and regulate them. As such, it seems we might need to find or create a route to a more direct exchange with the things with which we regularly interact.^6^

By appealing to the notion of answerability in human-to-human interaction, I propose to develop a technology-focused analog, which should be useful for conceptual and practical purposes in human–computer and human–robot interactions. Also, to be clear from the outset, I will not argue that we necessarily *should* hold technology responsible in this way. Rather, I mean to suggest only that, given the increasing ubiquity of sophisticated technologies in our daily lives and the fact that we might not be able to discern reasons for a system’s behavior, efforts to increase technology’s answerability might solve some problems, even if it creates others.^7^ That being said, I must clarify the nature of the problem at stake.

As legal and technical experts have acknowledged, algorithmic decision-making processes, particularly machine learning models such as artificial neural networks, are far from transparent (e.g. Arrieta et al. 2020; Kroll et al. 2017; Matthias, 2004). Many devices and programs arrive at their outputs by way of hidden layers of coding, and when human subjects are affected—for example, by being denied a bank loan, a job, or parole—there may be insurmountable barriers to receiving an answer as to why this event came about (Wachter et al., 2017). Human users and even the designers will simply not know why a system has arrived at a given decision. As Kroll et al. explain, machine learning systems “can update their model for predictions after each decision… Even knowing the source code and data for such systems is not enough to replicate or predict their behavior” (Kroll et al., 2017, p. 660). This is why, for example, an internet user’s website advertisements can change in real-time and entirely on their own. Demanding reasons for algorithmic outputs is often, by and large, a forlorn endeavor, even where such details are supposedly secured under data-protection laws (Wachter et al., 2017).

Many AI and robotic systems present us with potential problems in an immediate sense, then, by failing to meet demands for answers regarding their decisions and behaviors. But what exactly is the underlying problem of ostensibly mysterious technologies making decisions on our behalf? While many authors are quick to note the threats to our understanding and autonomy, a more complex puzzle is lurking here, one that finds an early articulation in Albert Borgmann’s (1984) notion of the ‘device paradigm’. According to Borgmann, we may be tempted to give-in to the allure of technology and its promise of alleviating our everyday burdens, such as preparing meals and repairing our belongings. What we thereby sacrifice, however, is a deeper understanding of and engagement with the world around us. As Borgmann puts it, we move more and more “from engagement to diversion” and with this move comes “feelings of loss, sorrow, and of betrayal” of our traditions and aspirations (1984, p. 105).

Similarly, recent thinkers help us to see that, because we develop and implement technological processes as *solutions* to our desire to automate laborious tasks, it might seem we should be content with our progress—or at least, our resulting ignorance and deskilling is the price to pay for greater efficiency and productivity (cf. Danaher, 2016b; Vallor, 2015). Should this lead us to pessimistic conclusions about our future with technology? Here is where Danaher’s argument for technological severance provides a fruitful framework for grasping the large-scale psychological problem at stake. The severance problem is presented as follows:

1. If humans are to live lives of flourishing and meaning, there must be some significant connection between what they do and what happens to them and the world around them.
2. The widespread availability of automating technologies severs the significant connection between what humans do and what happens to them and the world around them.
3. Therefore, the widespread availability of automating technologies undermines the capacity of humans to live lives of flourishing and meaning. (Danaher, 2019a, p. 102)

In support of the first premise, Danaher suggests that we cannot simply ‘sit back and enjoy the ride’—that is, we must maintain our ability to achieve things in the world.^8^ Here he appeals to Gwen Bradford’s work, which defends achievement as “a difficult process which culminates competently in a product” (Bradford, 2013, p. 205). But it is not entirely clear that products alone satisfy premise (1). Surely, there are significant connections between our actions and what happens to us—connections which are indeed being severed—but which cannot accurately be conceived in terms of creating products.

Accordingly, Danaher adopts a general view on Bradford’s notion, interpreting the products of our pursuits as including outcomes. For example, a completed marathon is certainly an achievement without being a product (Danaher, 2019a, p. 103); and so, the broader interpretation is quite appropriate. Still, when considering premise (2), namely the widespread availability of sophisticated technologies, it appears that the severed connections can be seen at a much more common and mundane level. Consider that smartphone users—particularly young adults—are relying more and more on their phones to get directions, buy basic products, and more.^9^ What we should take careful note of is simply the difference between undertaking these activities on one’s own—that is, without the use of smart technologies—and doing so with the ease of pressing a virtual button. Before the time of such technologies, we had to know our way around, or at least figure it out on our own; we had to go to stores to buy things; we had to remember phone numbers and birthdays, among other analog ways of life. But now, to a very large extent, we are indeed able to sit back and enjoy the ride. We can undertake the most basic activities passively, with the press of a button or a voice-activated command, or by letting automated processes completely take charge.^10^

This vision, I take it, represents the problem of technological severance. It is a feeling of loss or sorrow, or perhaps merely a subtle sense of disengagement from reality, when we rely more and more on the innovations that promise to make our lives better. If and when we reflect on the idea that technologies pose grave new challenges, or that they change us in ways we might not approve of, the extremities of solutions are to abandon it entirely or to embrace it so as to continue adapting to new, unfamiliar environments and hope for the best. My suggestion here is a modest one, namely that there must be a middle-ground, a way of harnessing technology’s benefits while retaining our connection to reality and things we care about. No doubt, for many it will be a substantial challenge to achieve such a balance. Just as the widespread use of sophisticated technologies severs the significant connections in our lives, the same sorts of technologies stand to sever the mundane, everyday connections between what we do and what happens to us.^11^ These latter connections, too, have an impact upon our flourishing and ability to find meaning. This can be seen with further elaboration on the threat to answerability and our everyday interactions with technology.

## The Value of Answerability

So far, I have delineated a key concept of responsibility in human-to-human interactions, namely the process by which we demand answers for others’ decisions and actions, and I suggested that this process is threatened in our interactions with some technologies. Indeed, at present, it may seem quite implausible—or simply strange—to demand answers even from the most sophisticated AI systems. I also outlined the severance problem and offered an expansion upon Danaher’s point that some technologies sever the significant connections in our lives; I take it that technology can also sever the more mundane connections. In this section, I explain further how the lack of answerability in our everyday interactions with technological devices constitutes a sort of severance. This, in turn, will help to show that our demands for answers from some devices are worth satisfying.

I want to begin by delving further into concepts of responsibility as seen in recent works in AI ethics. Consider the ‘relational’ approach provided by Coeckelbergh (2010, 2020). Here, much like with Shoemaker’s notion of answerability, the key to grasping a rich sense of responsibility is to look not only at the agent—namely, the one who acted, perhaps caused harm, and so on—but also to consider the role of those who demand answers. Coeckelbergh (2020) refers to this crucial party as the patient, following the term *moral patients* as those who are on the receiving end of moral treatment. When we investigate both roles together, we begin to see, as Coeckelbergh aptly suggests, that there is much more to responsibility than the traditional epistemic and control conditions handed down from Aristotle. Indeed, upon reflection, it seems that when we determine only who had sufficient knowledge and who was in control of bringing about an action or outcome, we address only the question of *agency*—that is, who knowingly and deliberately caused the action or outcome in question. But considerations of agency alone do not (yet) tell us exactly what makes that person *responsible*, in what ways they are responsible, and *to whom* are they responsible.^12^ After all, surely we can imagine cases (for example, involving children or psychopaths) where one knowingly and deliberately causes harm, but it is unclear how, to whom, or in what ways that agent may—or may not—be responsible.

Following Coeckelbergh’s suggestion, we must clarify not only the role of the agent but also the patient, the one who has been harmed (or benefitted, or generally affected), since responsibility is “relational and communicative” (Coeckelbergh, 2020, p. 2061). As stated by Coeckelbergh, “the agent needs to be able to explain to the patient why she does or did a particular action” (ibid: 2062). And this depiction appears quite accurate, namely in describing the process of answerability: the *agent* needs to be able to provide answers (reasons, motivations, etc.) to the patient. However, it is precisely here that we again run into difficulties when attempting to hold AI and robotic technologies responsible in this way. Although AI and robotic systems are ideally made to respond to our needs and commands, they cannot give answers—at least not in the form that may be demanded of them, not as “reasons” in the sense that humans have reasons.^13^ Thus, it seems that when we look specifically at the agent-patient relationship in cases involving technological systems, we lose sight of responsibility, which can be frustrating for users. In this way, technologies we interact with can sever our connection to world, namely by leaving us ignorant as to why things happen and why devices behave as they do. Granted, many of these events and behaviors will be ordinary and perhaps uneventful, but as I have suggested, our lack of understanding might be troubling nonetheless. Not only will it be psychologically unsatisfying for moral patients to receive no answers to their demands (cf. Danaher, 2016a), but the dialectic process itself is an extremely valuable interaction. As I explain, the process of demanding, giving, and receiving answers can be rightly considered a paradigm of a *moral responsibility exchange* (McKenna, 2012).

Coeckelbergh accepts that only humans can give reasons and, as a result, only humans can properly be responsible. No doubt, this is a highly intuitive assumption, one that is shared by many (e.g. Purves et al., 2015; Talbot et al., 2017). With this acknowledgement, Coeckelbergh maintains his focus on responsibility as a relation between an agent and patient, but effectively turns away from the agent and toward a proxy who might be able to *answer for* the technological system. Since only humans can give reasons, he says “responsible AI means that humans should get this task” (Coeckelbergh, 2020, p. 2064). Yet, we established that the sort of responsibility at stake is relational and communicative, specifically demanding answers from *the agent* in question. Hence, by shifting the locus of answerability to a system’s human associates—despite the intuitive nature of this move—we lose sight of the more meaningful, morally significant interaction that would take place directly between the agent and patient.

As an example of a more satisfying interaction, consider McKenna’s (2012) account of human-to-human responsibility as *conversational*. Here it is shown that the most paradigmatic moral responsibility exchange is one that takes place between the agent and the affected members of the moral community who then react. Specifically, in the first stage, the agent makes what McKenna calls a ‘moral contribution’, namely an action (or omission) which bears a morally significant meaning. The patient then responds by holding the agent responsible, initiating a dialogue—what McKenna call the ‘moral address’ stage. Then, in a stage of ‘moral account’, the agent has the opportunity “to extend the conversation by offering some account of her conduct, either by appeal to some excusing or justifying consideration or instead by way of an acknowledgement of a wrong done” (McKenna, 2012, p. 89).

By considering again answerability as a distinct and crucial responsibility mechanism, we can imagine that in the moral address stage, the dialogue initiated by the moral patient involves a demand for answers, and that the following stage then entails the agent’s opportunity to provide such answers.^14^ Where answers are inadequate, or where they are altogether impossible to obtain, moral patients find themselves at a loss as to why they have been affected and why the agents they interact with have behaved in questionable ways. At the same time, because interactive technologies are intended to meet our needs and do as we command, it may well seem that the agent herself plays a role in bringing about mysterious behaviors and outcomes. Due to a lack of answerability, then, technologies risk severing the connections between what we do—including how we interact with emerging systems—and what happens to us and the world around us. In this way, technology disrupts a key responsibility mechanism and thereby stands to undermine our ability to live flourishing and meaningful lives. If such systems, particularly those intended for regular and direct user interactions, were to meet our demands for answers, it may be that this threat of severance can be staved off. I turn next to such prospects.

## Technological Answerability

Given the notions of answerability employed above, along with the conversational model of responsibility I have presented as paradigmatic, it might seem that I am calling for AI and robotic technologies to behave and respond exactly as our fellow human beings would respond. However, I realize that this would be a hasty and perhaps forlorn request, and that there may be good reasons *against* designing sophisticated technologies in these ways, such as an increased propensity to deceive us. Nevertheless, I hope to have established several relatively modest claims by now. First, demanding and receiving answers is an important way in which we hold others responsible, and many—if not all—of today’s technologies with which we interact are incapable of engaging in such exchanges. Second, these processes are valuable as means of understanding the world, what happens in it, and how our interactions play a role in the events that come about. In short, we often want to understand, at least in rudimentary ways, why our technologies behave as they do; yet we cannot. So, instead, we must cede to a proxy, seeking answers from a system’s human associates. However, a direct exchange between agents and patients, even where the alleged agent is a technological system, would constitute a more robust picture of answerability. In this section, I outline what such an exchange might look like with some AI and robotic devices.

The notion of *technological answerability* I have in mind can be characterized as a capacity in technological systems for recognizing human demands for answers and responding accordingly. A full specification of technical features is beyond my purposes here, but it is clear that sensory components, such as sophisticated cameras and microphones, will be key to devices’ abilities to first receive inquiries and commands from human users. Likewise, advanced processors, artificial neural networks or other machine learning models, would need to train and refine answerability programs in their ability to identify and respond appropriately to the user with whom it is presently engaged. Additionally, a means of communicating the desired answers to users is needed, such as the already familiar sorts of audio responses (e.g. Siri) or via digital displays. Consider that recent work in AI has shown an increasing aptitude for some systems to recognize and learn from human emotions, reactions which can be properly considered morally significant, like anger, joy, and sorrow (cf. Marechal et al., 2019; Ren, 2009; Wang et al., 2016). I assume that these sorts of functions will continue to advance; but again, I do not maintain that they necessarily *should* advance and be widely deployed. All I want to claim here is that, if we value direct responsibility exchanges—even simple responses to our demands for answers—then we may have reason to build technological answerability into *some* systems in order to better facilitate engaged interactions and attentive usage of automated technologies. While answerability functions naturally must be developed, regulated, and utilized with the utmost care, as I explain further below, it is plausible at least in ideal scenarios that this mechanism stands to help individuals to better understand the technological processes at work around them, to resist the step from “engagement to diversion” (Borgmann, 1984), and thereby to retain a connection to the world and what happens in it. In this way, technological answerability is one potential response to the severance problem and, for that matter, worth exploring conceptually and perhaps also in practice.^15^ With this potentiality in mind, I should expand upon an important distinction that I noted at the outset.

Technological answerability, as described here, is a capacity for responding to human demands for answers—specifically, it is to respond *with answers* and not necessarily with *explanations* of a more technical variety. The distinction is key to our conceptual understanding of answerability and to its practical application in technology, particularly considering that full explanations entail much more than simple answers and, accordingly, are often taken to be an elusive goal in the design and regulation of AI systems. On one definition of *explainable AI*, Alejandro Arrieta and colleagues consider a system “that produces details or reasons to make its functioning clear or easy to understand” (Arrieta et al., 2020, p. 6). At first approximation, this notion seems to fit well with the idea of answerability outlined above. Yet, they also note that whether or not something is understood to a given user is itself difficult to determine objectively and may require a more rigorous sojourn into cognitive psychology. For these reasons, it is aptly proposed that in our attempt to make AI explainable, we would do well to think of explainability as being relative to a given audience.

The idea of relativizing explainability is echoed in recent accounts. As argued by Adrian Erasmus and colleagues, the *problem* of explainability, specifically in artificial neural networks, is largely due to the demand that such systems be “understandable to a non-specific and correspondingly broad audience” possibly including the general public (Erasmus et al., 2020, p. 26). However, it seems unreasonable to require that the inner workings of AI systems—such as the diagnostic tools making their way into healthcare—are understandable to the general public, particularly considering that these sorts of systems are often not fully understandable to developers who take part in their design. Rather than accepting the demand for widespread understandability, Erasmus and colleagues suggest that we can work to make systems like artificial neural networks *interpretable*, by which they mean we can produce explanations which are “in some way or another, *more understandable* than the explanation we began with” (ibid: 17, italics in original). And while this account helpfully draws close attention to the users and what they may be psychologically and cognitively capable of understanding, it appears that, like explainability, interpretability is still focused on what we can or want to obtain from the technological system itself. In responsibility terms, we are here still looking primarily at the moral agent, even when we consider the extent to which explanations of its behavior are understandable to a specific moral patient.^16^

In order to achieve a fuller grasp of responsibility, theorists like Coeckelbergh and McKenna encourage us to take seriously the perspective (and the demands) of the moral patient and, more broadly, the interactions and exchanges that occur between agents and patients. With this broader view in mind, I want to pivot away from the demand for explanations—or for audience-relative interpretations—and toward the demand for answers, which I take to be a wide class of responses that may be offered within a unique interaction.^17^ On the notion on technological answerability outlined here, what constitutes an adequate and potentially satisfying answer may well include explanations such as a system’s functionality. This is sometimes framed in terms of *ex ante* explanation, or transparency by design (e.g. Felzmann et al., 2020; Rossi & Lenzini, 2020). For example, we might design self-driving cars so as to maximize fuel-efficiency above all other factors. When a user then demands to know things like why the engine shuts-off at red lights, or why the acceleration is not as fast as other cars on the road, satisfactory explanations can refer to the initial design features. Answers might also include *ex post* explanation, namely details of specific algorithmic decisions, such as an individual’s data that featured in a given output. For example, we can demand to know whether or not irrelevant factors might have played a role in one’s loan application (cf. Wachter et al., 2017).

Beyond *ex ante* and *ex post* sorts of explanations, following McKenna’s description of the ‘moral account’ stage, an agent might give excuses, justifications, or even simply an acknowledgment of what happened. What is important to notice about these exchanges is that, depending upon the situation and the demands of the patient, a great diversity of responses could suffice to answer the demand for why some behavior was undertaken. That is, when it comes to human-to-human interaction, we can fulfill each other’s demands for answers by responding not only with explicit answers, but sometimes with excuses, justifications, or even simple acknowledgments of our actions and attitudes. Indeed, when we truly focus in on the variety of possible “answers” that may satisfy our demands, it appears that the range will be quite extensive and not limited to the precise reasons for which the agent acted. In other words, the process of demanding and giving answers is, at times, simply a pragmatic exchange, an interaction that takes place primarily to express and alleviate one’s concerns, or to create a sense of shared experience with others. Consider, for example, one friend asking another: “Hey, why do you always chew with your mouth open?” Cases such as these may well demand an answer, but not in the form of an exact reason or causal explanation. Indeed, technically accurate explanations may appear cynical, perhaps harmful to one’s relationship, even if humorous at times—consider the possible reply: “Because I need to breathe while eating.” Instead, we often want an agent to acknowledge her behavior, perhaps provide an apology or justification (e.g. “Sorry, I have a cold.”), or otherwise adjust the behavior according to the preferences of those with whom she regularly interacts.^18^

Similarly, select devices might be made to respond to individual users in ways that satisfy their individual needs and preferences. Just like in cases of human-to-human interaction, in some cases of human–computer interaction, we seek merely to understand what happened and why. Consider again the human user of an environmentally-friendly self-driving car and the desire to know more about its acceleration relative to other vehicles. In other cases, we may wish for an acknowledgment that what happened should not have happened and, accordingly, our demands for answers might be accompanied by a desire for things to transpire differently in the future. Given the state of machine learning systems and the ability of technologies to adapt to individual users, it is plausible to suppose that select devices could increasingly include interactive functions that satisfy an extensive range of human responses to a device’s behavior. Technological answerability, in this way, would be a wide-ranging and adaptable feature of sophisticated devices, one that could help us to understand why technology behaves as it does and that might more effectively meet individual user preferences.^19^

Consider a fictitious but conceivable scenario: a technologically-answerable personal AI program. Imagine that a music-playing app on my smartphone, such as YouTube, begins playing a new record from a band previously unknown to me, and that I find the new tunes awesome. No doubt, there are likely many causes lurking behind this appropriate match, some of which are indeed being displayed on these kinds of services—think of the Netflix recommendation categories that begin “Because you watched.” Still, it’s important to consider also that some of the causes behind the outputs given by such devices, such as corporate sponsors, remain more opaque to users but could be easily understood if offered as an answer. And this is the sort of mechanism I have in mind with respect to such applications. That is, on a technologically-answerable personal AI device, one could request information pertinent to the cause of the immediate output; and the information provided need not be a complex set of neuronal nodes through which a signal traveled in order to arrive at its output. Very often, we do not need—nor do we want—full *explanations*. We simply want answers. “Tell me why this new music was recommended,” I might demand. On the notion of technological answerability, the system would respond, perhaps vocally or via typescript, with the relevant causal information. “Because you seemed to like similar music in the past” or “Because the record label provided a sponsorship targeting listeners like you” and so on.^20^

Consider a second scenario: a technologically-answerable robotic assistant for the elderly. Imagine that a user notices her house lights being dimmed and that she is unsure as to why this happened. With the answerability mechanism outlined here, she could simply ask “Why did the lights go down?” and the assistant would respond with an answer, perhaps something like “You sleep better when nighttime conditions are initiated at 19:00.” Depending upon the user’s preferences, such an answer might satisfy her inquiry or invite further questions, and naturally the extent to which the system is able to continue responding will be determined by the state of the technology. The key mechanism suggested, however, is simply an ability to respond to demands for answers concerning immediate behavioral outputs. This sort of mechanism would help to retain the connections between what we do, including how we interact with and outsource tasks to smart systems, and what happens in the world around us.

Before turning to several closing qualifications, I must reiterate that technological answerability cannot, and likely should not, precisely resemble human answerability. The more descriptive claim—the fact that technology *cannot* do this—may come across as a limitation of the proposed functionality. Human answerability as I have outlined it here, is a morally-loaded notion, so to speak. It is a process whereby we make demands upon other moral agents, who can then offer something like reasons (including excuses and justifications) for their decisions, actions, attitudes, and so on. I do not claim that we can duplicate these sorts of features in our interactions with even the most sophisticated machines. But I also do not find it necessary for machines to truly be moral agents, or for their behavior to be motivated by human-like reasons, in order for us to have meaningful interactions with them. Rather, it seems that we can retain, or even enrich, our connection to the world by assuring satisfactory interactions with a host of diverse objects, whether humans or AI, or completely inanimate objects.^21^ As for the more normative claim—that technology likely *should* not replicate human answerability—surely, we must make efforts to guard against newfound harms of technology, like deception or unhealthy dependencies, among others. Still, on the account developed here, human answerability can serve as a model for desirable interactions. As I proposed at the outset, building a technology-focused analog to human answerability—even if noticeably different—might nonetheless help some users to better understand what happens, and thus to retain a connection to a world increasingly occupied by sophisticated devices and programs.

As I have been concerned to show here, there are reasons in favor of including in some systems a technological answerability function.^22^ In sum, our interactions with sophisticated technologies are increasingly common and such interactions undoubtedly play a role in noticeable events, including some of our more mundane experiences. We may often be at a loss and yet wish for basic answers as to why some behavior is undertaken. A world in which our understanding of why things happen has been completely lost is likely quite unsettling. But even where our ability to demand and receive answers is only slightly compromised may be unsatisfying, since our engagement in such interactions is a valuable process of locating responsibility. While exceptions must be made, as I turn to next, it seems that technological systems with which we regularly interact would be less likely to sever our connection to the world where they are able to satisfy our demands and help us to understand why things happen—that is, where they are designed for technological answerability.

## Qualifications: Many Possible Remedies, User-Relativity, and Discernible Differences

I began by noting that I wanted to explore and provisionally defend the idea of technological answerability, namely as a mechanism that lies between more extreme responses to the severance problem. Keeping with this agenda, several qualifications are worth highlighting as possible exceptions. First, the notion of technological answerability is meant to be one among many remedies worth exploring in response to the challenge of staving off the threats posed by sophisticated AI and robotic systems. Depending upon one’s preferences and experiences with technology, there will surely be other ways to maintain a connection to the world—such as periodically unplugging from technology, or adjusting one’s use of certain devices.^23^

Second and relatedly, designing devices so as to successfully respond to our demands for answers will, of course, be more or less satisfying relative to the user in question, the device, and the wider context in which the interactions occur. As we can imagine, for some users, the explanations of a product’s functionality may be too superficial for them to retain a truly meaningful connection to the world.^24^ Accordingly, it seems crucial to undertake efforts at understanding, measuring, and evaluating the impacts of various modes of human–computer interaction, including the effects of answerability mechanisms. Some of the contemporary models of interdisciplinary socio-technical research (e.g. McLennan et al., 2020) might help to assess whether or not answerability is a beneficial feature of certain devices, and if so, for which kinds of users. Additionally, depending upon what we learn from such research, governments and electorates can make efforts at implementing any necessary safeguards in local and international regulations. Just as measures like the EU’s General Data Protection Regulation are emerging to address concerns for data privacy and security, we can hope that societal mechanisms will become better equipped to promote our psychological wellbeing in light of our increasingly technological environments.

Finally, some will likely object to my account with the thought that, even if answerable technologies help us in some ways, the risks of harm are too great. For example, some users may become *further severed* from the world if their devices are better able to deceive, effectively decreasing users’ understanding and autonomy, and removing them from decision-making processes (cf. Boden et al., 2011; Bryson, 2018; Theodorou et al., 2017; Van Wynsberghe & Robbins, 2019). Certainly, this concern must be taken seriously, and in fact, doing so further highlights the importance of my initial qualifications—namely that there may be other ways to retain our understanding, and that for some users, technological answerability might be unsatisfying or even dangerous. Here again I also emphasize that technological answerability need not entail exact replication of human answers. To be sure, the idea of technologically-answerable AI and robotics is fully consistent with efforts to ensure discernible differences between human and technological responses, so as to help protect against the harms of deception. No doubt, future research and regulatory measures will need to be implemented in the service of assuring our wellbeing as we develop and possibly employ novel interactive features of emerging technology.

## Conclusion

In closing, I want to step back and briefly address two broad lines of thought that my account appears to raise, specifically concerning responsibility generally and then the potential for staying connected to a world increasingly populated by technological systems.

Consider first that holding one another responsible—and holding ourselves responsible—can be seen as a crucial mechanism (or set of mechanisms) by which we establish, communicate, and reinforce our demands for moral regard, and by which we participate together in a shared interpersonal community. Within our relationships and interactions, we evaluate the behavior and attitudes of others, and of ourselves, and very often such processes are either implicitly or explicitly an attempt to understand others and to improve the future. It is only natural that we respond to negative events in ways that might decrease their future occurrence—consider punishment or expressions of anger—and to positive events in ways that might encourage their recurrence—consider expressions of gratitude. Yet, among the key components needed for our responses to be successful, or even sensible, is a capacity in the target agent to hear and understand us, and to respond to our demands appropriately. When such capacities are not possessed by the target, or where they are atypical in some way, we make adjustments to our manner of interaction. As I noted above, and as many responsibility theorists observe, this helps to see why we do not—and why we should not—hold children or psychopaths (among others) responsible in the same ways we hold fully functional adults responsible. Nonetheless, our interpersonal lives are highlighted and enriched by interactions with a great diversity of others, and importantly, we find ways of understanding each other and perhaps improving the future.

Technological devices and programs, in many ways, have entered into this diverse set of others with whom we regularly interact. Our interpersonal communities are changing, and for this reason, it seems only natural that we seek out newfound ways of understanding each other and improving our future together. Note here that the question at stake does not necessarily concern how, if at all, we can admit highly atypical agents into the natural moral community (cf. Tigard, 2021b). Whether we grant AI and robotic companions, for example, a certain moral status—like agency, patiency, or newfound rights and duties—can be addressed separately from the question of how we might interact with them so as to retain our understanding of the world and connection to it. Concerning the latter inquiry, what I have offered here is simply one among many possible ways of interacting with sophisticated technologies, which might help us to continue adjusting to our changing interpersonal environments.

Lastly, consider again that the backdrop to my inquiry was a potential threat to our wellbeing posed by emerging technology. Our potentially increasing severance from the world and what happens in it, however, is merely one of the difficulties we must keep in focus, and indeed many possible remedies to the severance problem are likely to come into conflict with other reasonable perspectives on our relationship with technology, leaving us with complex tradeoffs to consider. As surveyed above, it may seem best to simply unplug from technologies that obscure our understanding and connection to the world—but then we miss out on the gains in efficiency, productivity, and comfort offered by technology. For others, it will seem that we should fully embrace the benefits of emerging technologies, and any disconnection from reality can be remedied by exploring alternative, virtual realities. But there, no doubt, a host of other challenges arises, such as how we should create and regulate these new environments, and how exactly we can assure our continued wellbeing in the analog world we leave behind.

The moderate path outlined here does not call for abandoning today’s technologies, nor does it entail diving deeper into virtual realities. I believe there are ways of harnessing some of the devices and programs that promise to improve our lives, and ways of doing so while staying connected to the world and retaining our understanding of what happens in it—at least to the extent that fits our individual preferences. That is, some will surely not notice any sort of severance, and others, even if they notice, will not care to stave off the threat. But I assume there are others like me, who notice that emerging technologies are changing us and the ways we interact—with each other and with the world—and who want to assure that with those changes we do not lose sight of how and why things happen, even concerning the tasks we choose to outsource to technology. Still, I readily admit that the idea of technological answerability bears numerous challenges of its own, some of which are technical, others legal, and still others revealing serious moral concerns. The potential for deception, and possibility of being further severed from what happens in our technological world, again must be taken seriously. At the same time, for those who value responsibility in our interactions with others, including the AI and robotic systems increasingly occupying our everyday lives, it will be worthwhile to explore new ways of holding technology answerable.

## Footnotes

1 Note that the demand stated here is for *answers*, not necessarily for *explanations*, which I take to be more technically-robust and often impossible, considering the now notorious “black box” problem. This distinction is further clarified below.

2 The term can be traced back to theories of moral sentiments developed by Adam Smith and David Hume. In its contemporary form, the notion follows most notably from P.F. Strawson (1962).

3 While legal notions (like liability) are important to update in light of technological developments, I leave them aside here and suggest legal scholarship addressing such challenges—e.g. Smith and Fotheringham (2020), Sullivan and Schweikart (2019), Wachter and Mittelstadt (2019).

4 See Köhler et al. (2017) for arguments against ‘responsibility gaps’ focused exclusively on accountability.

5 Nonetheless, it is worth noting that human agency is a vastly diverse. Considering that our practices of holding each other responsible are widely adaptable to natural forms of agency, it seems we might likewise be able to adapt to artificial forms of agency. See Tigard (2021a).

6 Some readers will be familiar with Mike Judge’s 1999 movie *Office Space*. Picture the iconic scene where three disgruntled office workers take the copy machine to a field and passionately destroy it, as if exacting years of pent-up revenge for the pain it brought them. As I see it, this illustrates that our actions and attitudes can be elicited by and directed at machines in ways they couldn’t be by machines’ creators, at least not in similarly satisfying (or similarly humorous) ways.

7 Here I should emphasize a potential strength of my account. By developing a mechanism by which we might locate a key type of responsibility in technology, the account offered here addresses the technological severance problem, as I show, but also the concerns for a “responsibility gap”. For expansion on the latter, see Tigard (2020).

8 For a recent expansion of this view, see Danaher and Nyholm (2020). Also important to note is Danaher’s (2019a) framing of a flourishing and meaningful life, namely in terms of one’s contributions to the Good, the True, and the Beautiful—representing moral, scientific, and artistic endeavors. While these are very plausibly key aspects of flourishing and meaningful lives, as I explain, I also have in mind the more mundane connections threatened by technology.

9 See the 2016 Pew Research report: https://www.pewresearch.org/fact-tank/2016/01/29/us-smartphone-use/

10 Relatedly, see Danaher (2019b). My concern for our increasing passivity in mundane tasks closely resembles Danaher’s worries about the state of future humans as depicted in the movie *Wall-E* (Danaher 2019a, p. 87).

11 Note that I am interpreting Danaher’s second premise broadly, to include the significant connection between what humans do—that is, with their use of technology, as well as what technology does for them—and what happens to them and the world around them.

12 Although agency and responsibility are often treated simultaneously, in contemporary ethics literature they usually remain distinct concepts, even if commonly related. For example, many hold that *responsible* agency is a subset of agency simpliciter (McKenna 2012, p. 12).

13 Support for this view is found, e.g., in Purves et al. (2015) and Talbot et al. (2017). For a contrasting account, in favor of robots acting for reasons, see Frank and Nyholm (2017).

14 It should be noted that McKenna does not subscribe to Shoemaker’s pluralistic view of responsibility, wherein answerability is one among three distinct types. In fact, there are stark differences between these authors, but which cannot (and need not) be elaborated here.

15 I claim that my account represents *one potential response*, considering that there are likely numerous ways of maintaining a connection to the world. Indeed, for some, implementing technological answerability might risk further severance, say, by encouraging diversion or even deceiving us. I return to such issues below.

16 Granted, the efforts of Erasmus et al. (2020) are not directed at filling out an account of responsibility, as I am concerned to do here. Thus, my appeal to their work is not so much a critique, but rather a means of highlighting a useful understanding of *explanations*, namely as a contrast to *answers*, to which I now return.

17 Aside from the narrow agential focus in the demand for explanations, another reason to leave it behind is that full explanations are often impossible to obtain even from humans, say, due to implicit biases or post-hoc rationalization (cf. Doris, 2015; London, 2019).

18 If the demand for answers is satisfiable on a purely pragmatic basis, it would seem that many satisfactory answers will not be complete or entirely honest. While this would be problematic in cases of deliberate deception, as I address in conclusion, it may simply be a peculiar feature of human psychology (and of linguistic conventions) that sufficient “answers” may be indirect, incomplete, inaccurate, and so on. I thank an anonymous reviewer for prompting this reflection.

19 That being said, I do not assume that all users of answerability-enabled devices will prefer utilizing this sort of functionality. Certainly, some users will not care to better understand reasons for a system’s behavior or, in some cases, may not care (or notice) that the connection is being severed between what they do and what happens in the world. I return to this concern in my qualifications below. For insightful discussion that invokes a related line of thought arguing against principles requiring explicability for AI, see Robbins (2019).

20 Granted, there are a host of technical and legal questions to be raised here, along with useful technical and legal supplements. For example, under the EU’s GDPR, it may be enforceable to follow the response with a demand to remove one’s personal data that led to the output in question. Given spatial limitations, I must leave aside these additional considerations, as my main concern has been to show the moral relevance of technological answerability.

21 Consider, for example, a person enriching their life—say, non-trivially improving their moral character—via experiences with artwork or with nature.

22 For a related position supporting ‘socially responsive’ technology, see Tigard et al. (2020). With its focus on answerability, the present account can be seen as a more specific realization of the idea of social responsiveness in technology.

23 Perhaps becoming more virtuous with respect to specific devices and apps (e.g. social media), or with respect to our increasingly technological world generally, will help to promote one’s wellbeing (Vallor, 2016).

24 I thank an anonymous reviewer for helpful comments wherein this line of concern was raised.
